# OCTN1 (SLC22A4) as a Target of Heavy Metals: Its Possible Role in Microplastic Threats

**DOI:** 10.3390/ijms252313218

**Published:** 2024-12-09

**Authors:** Luana S. Brunetti, Mariafrancesca Scalise, Raffaella Scanga, Lara Console, Michele Galluccio, Mauro F. La Russa, Lorena Pochini, Cesare Indiveri

**Affiliations:** 1 Department DiBEST (Biologia, Ecologia e Scienze della Terra), University of Calabria, Via Bucci 4C, 12B, 87036 Rende, Italy; luanabbiologia@gmail.com (L.S.B.); mariafrancesca.scalise@unical.it (M.S.); raffaella.scanga@unical.it (R.S.); lara.console@unical.it (L.C.); michele.galluccio@unical.it (M.G.); mauro.larussa@unical.it (M.F.L.R.); 2 National Research Council (CNR), Institute of Biomembranes, Bioenergetics and Molecular Biotechnologies (IBIOM), Via Amendola 122/O, 70126 Bari, Italy

**Keywords:** SLC22A4, heavy metals, liposomes, acetylcholine, pollution

## Abstract

Microplastics represent a threat due to their ability to enter the food chain, with harmful consequences for living organisms. The riskiness of these particles is also linked to the release of other contaminants, such as heavy metals. Solute Carriers (SLCs) represent eminent examples of first-level targets of heavy metals due to their localization on the cell surface. Putative targets of heavy metals are the organic cation transporters that form a sub-clade of the SLC22 family. Besides the physiological role in the absorption/release of endogenous organic cations, these transporters are crucial in drug disposition and their interaction with xenobiotics. In this work, the human SLC22A4, commonly known as OCTN1, was used as a benchmark to test interactions with heavy metals released by microplastics, exploiting the proteoliposome tool. The potency of metals to interfere with the OCTN1 function has been evaluated by measuring IC50 values calculated in the micromolar range. The molecular mechanism of interaction has been defined using site-directed mutagenesis and computational analyses. Finally, some chemical and physiological thiol-reacting compounds show the capacity to rescue the metal-inhibited OCTN1 function. The conclusions drawn on OCTN1 can be extended to other members of the SLC22 family and orthologous transporters in fish.

## 1. Introduction

Plastic pollution represents one of the global threats to living organisms, with both seas and oceans being ideal basins for accumulating great amounts of these contaminants. Indeed, islands of plastic waste have grown over the years in marine environments, reaching more than 250 K tons of total plastic [1]. The danger of plastic waste in the environment is related to several factors, including their physical, mechanical, and chemical features. In this respect, increasing efforts are underway to accomplish a systematic classification of plastic particles according to their size and composition, and currently, three main categories have been defined (Table 1) [2]. 

When looking at living organisms as a target, microplastics are those raising major concerns because their small size facilitates ingestion and accumulation in the entire food chain, including humans [3,4]. Furthermore, microplastics are vehicles of other contaminants with unpredictable global effects on metabolism, cell proliferation, and even more intricate and complex interactions with entire organisms [5]. Therefore, great efforts are needed to fully characterize the collection of potential threats linked to the worldwide spread of microplastics. In this respect, appropriate biomarkers for monitoring the harmful effects of plastic pollution are needed in living organisms, including humans.

### 1.1. Microplastics as Carriers of Heavy Metals 

In this very large and open field of investigation, several studies highlighted the possibility for microplastics to accumulate and release heavy metals [6,7,8,9], which are absorbed during the production process or by their interaction with other pollution sources [10]. Generally, the release of metals follows kinetics linked to the diffusion of the metals themselves inside the plastic particles and depends, besides other parameters, on microplastic diameters and porosity, pH, the salinity of the medium in which they are found, and solar radiation. Moreover, gastrointestinal fluids of aquatic animals can digest microplastics and, hence, release heavy metals [11,12]; it has been demonstrated that ingested microplastics can release heavy metals in human tissues as well [3,13]. Then, microplastics represent another source of heavy metals, which are well known as extremely dangerous for all living organisms. Indeed, these toxicants can accumulate in tissues with mechanisms known as biomagnification [14,15]. Once in cells, some heavy metals can form metal–thiol bonds with cysteine residues of proteins, altering their stability/mobility and functionality in cells. The wide range of negative effects of heavy metal contamination is related to the sizable number of their molecular targets and, hence, cell pathway alterations. Among the biological targets of heavy metals, there are membrane proteins belonging to the SLC superfamily, which contain an average of 11 cysteine residues in their structure and at least 1 cysteine residue in each protein, with only one exception [16]. 

### 1.2. OCTN1 (SLC22A4): A Benchmark for Studying Interactions with Heavy Metals

In line with previous data, the fourth member of the SLC22 family, namely OCTN1, is an acknowledged target of mercury-derived compounds [17]. OCTN1 can also recognize several drugs and, hence, contribute to their delivery to human tissues [18]. For these reasons, SLC22 proteins are included in the FDA guidelines as major players in pharmacodynamics and drug disposition [19]. OCTN1 has been described as a poly-specific transporter in ex vivo [20] and in vitro [21] models, given its ability to recognize both cations or zwitterions as substrates with different mechanisms. Indeed, this transporter mediates the uptake and the efflux of physiological cations such as acetylcholine and choline in a sodium-independent fashion [21]; at the same time, OCTN1 can recognize the fungi metabolite ergothioneine and the vitamin-like cofactor carnitine with a sodium-dependent mechanism [22,23,24]. Recently, the two independent pathways for cation/zwitterion binding and translocation were investigated by in silico and in vitro strategies [23]. 

### 1.3. OCTN1 in the Non-Neuronal Cholinergic System 

This broad substrate specificity highlights various roles in cell physiology, from the absorption of the antioxidant ergothioneine and carnitine to the release of acetylcholine with anti-inflammatory action in the non-neuronal cholinergic system (NNCS) [19]. Furthermore, the low-affinity carnitine transport can compensate for the lack or misfunction of the high-affinity carnitine transporter OCTN2 [20,23]. In addition, OCTN1 is involved in the disposition of carnitine or its derivative acetyl-carnitine when pharmacologically administered as neuroprotectants [25,26]. This evidence links the OCTN1 expression and function to the RSST (Remote Sensing and Signalling Theory) [27,28] as a player of the responsive/adaptive system allowing inter/intra-organ (gut–brain and gut–kidney axis) and inter-organismal communication. In good agreement, NNCS is active in all kingdoms of life due to the role of acetylcholine in the control of basic cell functions [29,30]. Finally, the involvement of OCTN1 in chronic inflammatory diseases and metabolic disorders suggests its role in cell senescence and ageing processes characterized by the so-called “inflammaging” phenotype [31,32]. Moving from the above-described premises, in this work, we aim to employ the human OCTN1 as a benchmark to study the effect of heavy metals potentially released by microplastics after ingestion by fishes and, therefore, by humans. In this research, OCTN1 could represent one of the food chain rings affected by microplastic pollution; indeed, OCTN1, which evolved with vertebrates, has orthologs in species other than humans, among which fishes also enter the human food chain [33,34]. Therefore, unravelling the molecular mechanism of heavy metal–OCTN1 interactions could add important information for explaining and, hence, preventing or treating the effects of heavy metal pollution in humans. 

## 2. Results and Discussion 

The heavy metals reported in Table 2 were identified in microplastics sampled in six stations of the Calabria Region coast, using qualitative and quantitative approaches. In brief, Ionian and Tyrrhenian sites were sampled, and microplastics were analyzed in terms of their size, shape, colour and metal content with the following relative abundance: Lead > Chromium > Zinc > Cadmium. These findings are in good agreement with an already published study on the release of heavy metals from microplastics in the marine environment [12]. 

As mentioned above, OCTN1 can be considered an ideal biological candidate in the context of environmental toxicology studies due to several important features: (i) it is a cell membrane protein being considered a first-level target for xenobiotics [35]; (ii) it harbours in its structure seven cysteine residues, which can be targets of mercury [17]; (iii) it plays multiple functions in humans, mediating the absorption/release of physiologically relevant molecules [19]; (iv) it has orthologues in different vertebrates including fishes, such as *Danio rerio* [33] and *Oncorhynchus mykiss* [34], which is one of the most common cold-water fish species in aquaculture and the seventeenth widely cultivated commercially important finfish in the world and, hence, in the human diet [36]; and (v) it is also a drug transporter, hence, the impairment of its function may also affect drug distribution and response in humans and other animals. It is noteworthy that even if exposure to heavy metals is a well-known human threat [17] (https://www.who.int/publications/i/item/WHO-FWC-PHE-EPE-16.01-eng (accessed on 21 October 2024)), it acquires more relevance for global health since the concept of the exposome emerged [37] (WHO). Indeed, considering an “exposome” referring to the totality of exposures to internal and external factors, the widespread distribution of heavy metals, together with their strong ability to interact with macromolecules, can be considered an emblem in such a continuously evolving research area. 

### 2.1. Reconstitution of Human OCTN1 in Proteoliposomes and Effects of Heavy Metals

**Figure 1:**
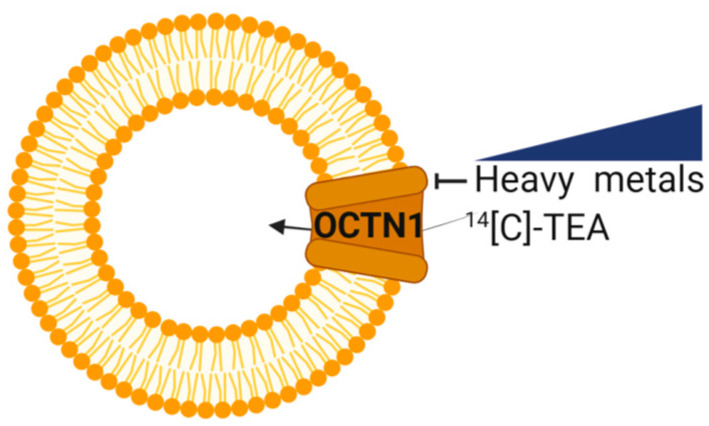
Inhibition of [^14^C]-TEA transport mediated by OCTN1 in the presence of heavy metals. The experimental model is depicted as a sketch. In orange the protein OCTN1; the arrow indicates the direction in which OCTN1-mediated [^14^C]-TEA transport occurs. The crossed-out line indicates inhibition of [^14^C]-TEA transport by heavy metals (blue).

Moving from these premises, the effect of Hg^2+^, Cd^2+^, Cr^2+^, Pb^2+^, and Zn^2+^ found to be released by microplastics was evaluated on the human OCTN1 using the well-characterized experimental model of proteoliposomes (Figure 1) in which the effect of Hg^2+^ was previously established [17].

**Figure 2:**
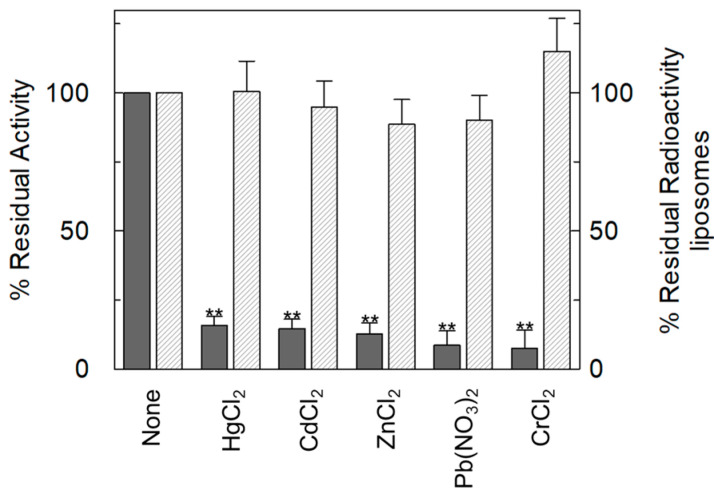
The inhibition of [^14^C]-TEA transport mediated by OCTN1 in heavy metals. The protein reconstitution procedure is described in the Section 3. The uptake started with the addition of 0.1 mM [^14^C]-TEA to proteoliposomes in the presence of 250 µM of indicated metals. The transport was stopped after 60 min according to the stop inhibitor method. Percent residual activity with respect to the control (without heavy metals addition) is reported (dark grey bars). To the right of the y-axis, data collected on liposomes without incorporated proteins are reported as the percentage of residual radioactivity associated with liposomes (white bars) due to diffusion/association with the vesicles with respect to the control (liposomes without heavy metals addition). The amount of radioactivity detected in liposome controls was no higher than 15% with respect to that which entered proteoliposomes harbouring OCTN1 in the membrane. The values are the means ± SD from three independent experiments. Data in the presence of the heavy metals were significantly different from the control (without heavy metals addition), as estimated by Student’s *t*-test (** *p* < 0.01).

This experimental system successfully mimics the cell conditions since OCTN1 is inserted in the artificial membrane with the same orientation as in the native membrane, i.e., with the internal and external compartments of liposomes corresponding to the internal and external environment of cells. This allows reliable information on the specific effects of the toxicants on the single protein included in the membrane to be obtained (Figure 1). In this system, the effect of heavy metals was tested on the uptake of radiolabelled TEA (Tetraethylammonium), i.e., the well-known and widely used prototype of a cationic substrate of OCTN1 [38]. The addition of divalent metals strongly inhibited the uptake of [^14^C] TEA in proteoliposomes (Figure 2). It has to be stressed here that our experimental model, which is exquisitely in vitro, is intended as a proof-of-concept approach devoted to the identification of a molecular target of environmental pollutants, such as heavy metals; therefore, the used concentration in these experiments was intentionally high, as an initial screening, assuming the condition of a high-polluted environment or a biomagnification phenomenon in tissue, which is a common feature for heavy metals [14,39,40,41].

**Figure 3:**
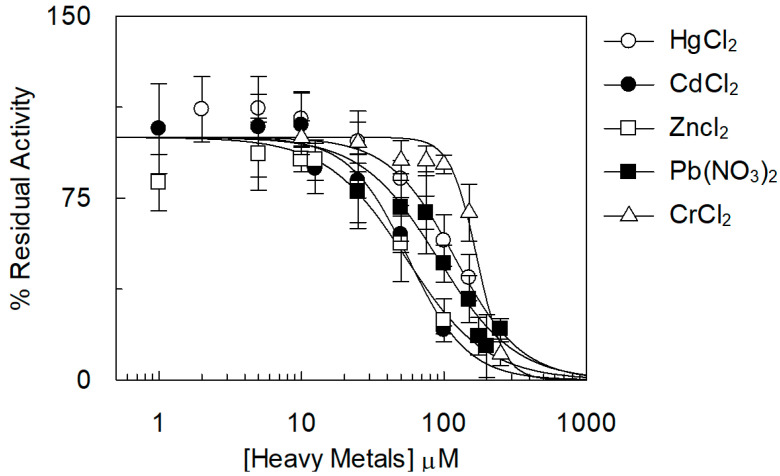
Dose–response analysis of the inhibition of the [^14^C]-TEA transport by heavy metals. The reconstitution was performed as described in the Section 3. The transport was started by adding 0.1 mM [^14^C]-TEA to proteoliposomes together with the indicated concentrations of HgCl_2_ (empty circle), CdCl_2_ (filled circle), ZnCl_2_ (empty square), Pb(NO_3_)_2_ (filled square), and CrCl_2_ (empty triangle), and ended with the stop-inhibitor method after 60 min. The figure shows the residual activity as a percentage compared to the control (without heavy metals addition). The values are means ± SD from three independent experiments.

To evaluate the potency of inhibition, dose–response analyses were performed (Figure 3). 

As usual for potent inhibitors, the measured IC_50_ values were in the micromolar range, i.e., from 70 to 200 µM (Table 3). It is not trivial to consider that these concentrations are lower than those locally reached in the organism upon microplastic ingestion due to the release mechanism, which depends on several mechanisms, some of which are still not completely explained [42]. 

### 2.2. Role of OCTN1-Cys Residues in the Interaction with Heavy Metals 

**Figure 4:**
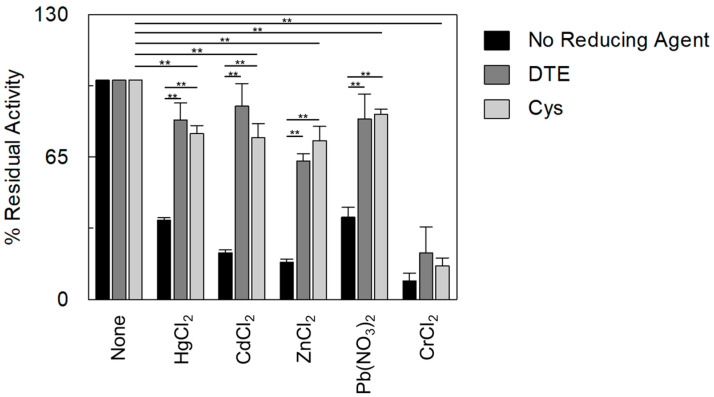
Effects of DTE and Cys on the inhibition of [^14^C]-TEA transport mediated by OCTN1 with heavy metals. The protein reconstitution procedure is described in the Section 3. The uptake started with the addition of 0.1 mM [^14^C]-TEA to proteoliposomes together with 100 µM HgCl_2_ or CdCl_2_ or ZnCl_2_ or Pb(NO_3_)_2_, or 250 µM CrCl_2_ in the absence of reducing agents (black bars) or in the presence of 5 mM reducing agents; DTE or Cys are indicated by dark grey or light grey bars, respectively. The uptake ended with the stop-inhibitor method after 60 min. The percentage of residual activity with respect to the control (without heavy metals addition) was reported. The values are means ± SD from three independent experiments. They were significantly different from samples without reducing agents and from the control (without heavy metals addition), as estimated by Student’s *t*-test (** *p* < 0.01).

As stated in the introduction, heavy metals are known to interact with cysteine residues of the target proteins via the formation of metal–thiol bonds. Some canonical binding sites for metals have been described, such as the CXXC, CXXXC, and MxCXXC motifs [16]. These are found in different proteins, i.e., intracellular targets and membrane proteins, including those responsible for the direct transport of heavy metals in cells [43,44,45]. Interestingly enough, non-canonical binding motifs are also expected to exist based on the spatial positioning of close thiol residues according to the 3D structure of protein microdomains [46]. This property, which cannot be identified in the primary structure of proteins, further explains the wide toxicity exerted by heavy metals [46]. It has to be stressed that a specificity issue arises from the available data in the literature, considering that the same signature may recognize different metals [47]. OCTN1 contains vicinal thiol residues, which are not adjacent in the protein sequence, forming microdomains previously identified as targets of Hg and Hg-derivatives by site-directed mutagenesis and in vitro transport assays [17]. Therefore, we hypothesized that the effects described in Figure 3 and Table 3 are ascribable to the interactions of the tested heavy metals with the cysteine residues of OCTN1 via the formation of metal–thiol bonds. To support this hypothesis, the effect of physiological and not physiological-reducing reagents was evaluated on the inhibition exerted by heavy metals (Figure 4).

Indeed, these reagents can cleave, in most cases, the metal–thiol bond [17]. In good agreement with the proposed role of OCTN1-Cys residues, the addition of DTE and cysteine prevented the inhibitory effects of all the tested divalent cations with the only exception of Cr^2+^, the ability of which to interact with residues other than Cys has been described [48]. It has to be stressed that four out of the seven Cys residues of OCTN1 are located in the large extracellular loop between TM1 and TM2, making them an ideal target(s) for xenobiotics. 

### 2.3. Computational Analyses on hOCTN1 Homology Model

**Figure 5:**
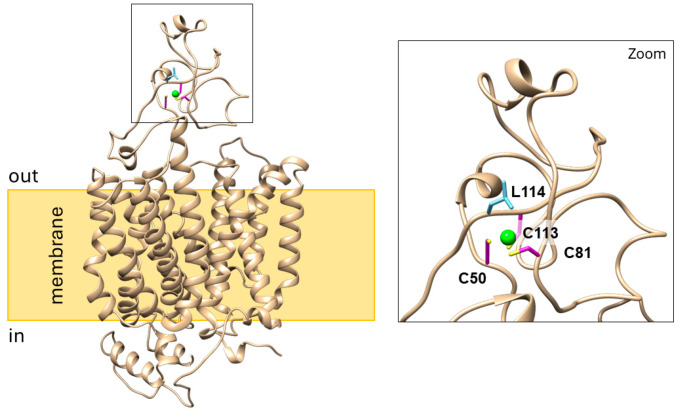
Lateral view of the homology model of human OCTN1 showing the predicted Cadmium binding site. The 3D structure of OCTN1 is shown with ribbon representation (sand). Cadmium is in green; the amino acids involved in the binding are depicted in magenta/yellow (C50, C80, C113) and Cyan (L114). Molecular graphics and the visualization of MIB2 results were performed with the UCSF Chimera v.1.14 software (Resource for Biocomputing, Visualization, and Informatics, University of California, San Francisco, CA, USA).

Then, computational analyses were performed to support experimental data: the homology model of human OCTN1 was built based on the CryoEM structure of human OCT3 [23] and analyzed using the MIB2 tool for predicting the binding template of Cd^2+^, i.e., the most effective among the tested metals (Table 3). The MIB2 tool constructs metal ion-binding templates that consist of a protein spatial region of 3.5 Å around the ion. These regions are selected from 3D structures containing ions deposed in the Protein Data Bank (PDB) or predicted by AlphaFold. Then, the structure to be analyzed (such as OCTN1) is locally screened against the ion-binding templates, as previously described [49]. As shown in Figure 5, the best-scored Cd^2+^ binding site on the outward-facing part of OCTN1 includes Cys50, Cys81, Cys113, and Leu114 residues. 

### 2.4. Analyses of Cys-Less Mutant of the Human OCTN1 Reconstituted in Proteoliposomes

To definitively prove the involvement of OCTN1 Cys residues in interactions with metals, another strategy was adopted using an OCTN1 mutant in which all cysteine residues were mutated to alanine (Cys-less OCTN1). 

**Figure 6:**
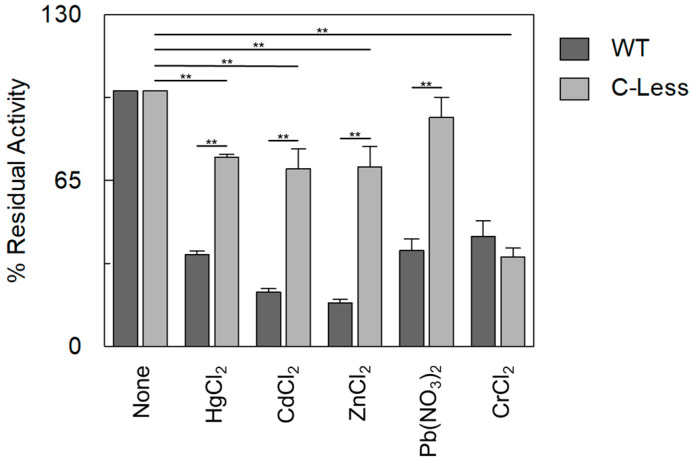
The inhibition of [^14^C]-TEA transport mediated by OCTN1 WT and C-less in the presence of heavy metals. The reconstitution procedure of WT (dark grey bars) and C-less (light grey bars) is described in the Section 3. The uptake started with the addition of 0.1 mM [^14^C]-TEA to proteoliposomes in the presence of 100 µM HgCl_2_ or CdCl_2_ or ZnCl_2_ or Pb(NO_3_)_2_, or 250 µM CrCl_2_. The transport was stopped after 60 min according to the stop-inhibitor method. The percentage of residual activity of OCTN1 WT and OCTN1 C-less with respect to the control was reported. The values are means ± SD from three independent experiments. Significant differences in C-less were obtained with respect to the control (without heavy metals addition) and C-less with respect to OCTN1-WT, as estimated by Student’s *t*-test (** *p* < 0.01).

**Figure 7:**
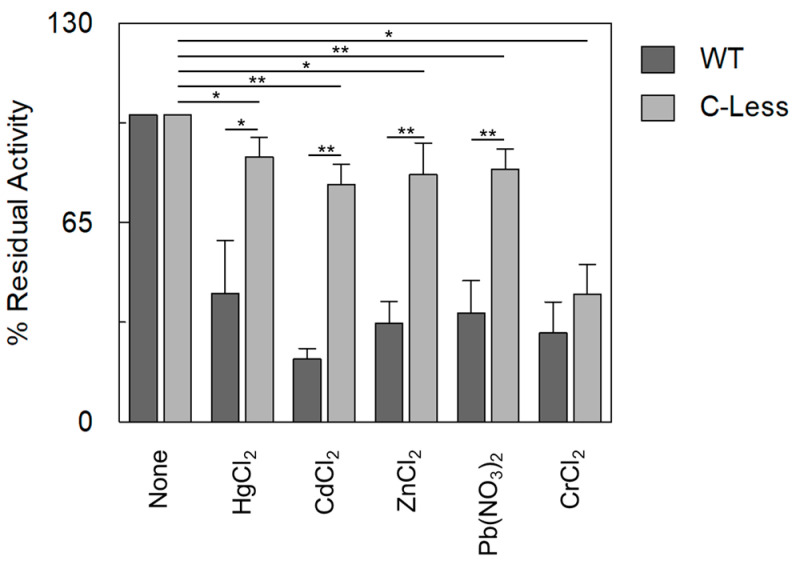
The inhibition of [^3^H]-Ach transport mediated by OCTN1 WT and C-less in the presence of heavy metals. The WT (dark grey bars) and C-less (light grey bars) proteins were reconstituted as described in the Section 3. The transport was started by adding 0.1 mM [^3^H]-Ach to proteoliposomes in the presence of 100 µM HgCl_2_ or CdCl_2_ or ZnCl_2_ or Pb(NO_3_)_2_, or 250 µM CrCl_2_. The transport was stopped after 60 min according to the stop-inhibitor method. The percentage of residual activity of OCTN1 WT and OCTN1 C-less with respect to the control was reported. The values are means ± SD from three independent experiments. Significant differences in the C-less were obtained with respect to the control (without heavy metals addition) and of C-less with respect to OCTN1-WT, as estimated by Student’s *t*-test (* *p* < 0.05; ** *p* < 0.01).

The data reported in Figure 6 show that the Cys-less OCTN1 was virtually not affected by heavy metals; the residual-measured inhibition can be ascribed to the presence of other residues, such as His, whose ability to coordinate heavy metals is well acknowledged [50]. The only exception was again Cr^2+^, confirming that the inhibitory effect on OCTN1 may be due to a molecular mechanism involving residues other than cysteine [48]. To better evaluate the effect of heavy metals on the physiological transport properties of OCTN1, the physiological substrate acetylcholine was also tested on WT and Cys-less OCTN1 (Figure 7). 

Overlapping results were obtained with respect to TEA, further demonstrating that OCTN1’s functionality is altered when assaying a physiological substrate. 

### 2.5. Acetylcholine-Mediated Transport of OCTN1: Inhibition and Link with NNCS

**Figure 8:**
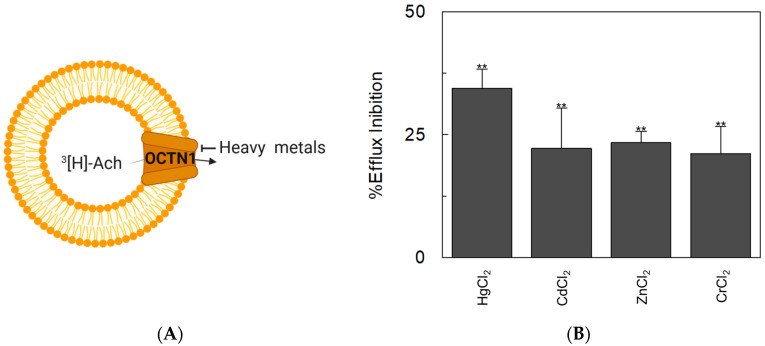
The inhibition of [^3^H]-Ach efflux mediated by OCTN1 WT in the presence of heavy metals. (**A**) The experimental model is depicted as a sketch. In orange, the protein OCTN1; the arrow indicates the direction in which OCTN1-mediated [^3^H]-Ach transport occurs. The crossed-out line indicates inhibition of [^3^H]-Ach transport by heavy metals. (**B**) The protein reconstitution procedure is described in the Section 3. The uptake of 0.1 mM [^3^H]-Ach was performed in 120 min. Then, the efflux of [^3^H]-Ach was measured in the presence of 100 µM indicated heavy metals. The efflux was stopped after 60 min according to the stop inhibitor method. Data were calculated as the percentage of efflux inhibition with respect to efflux without externally added heavy metals. Results are the mean ± SD of three independent experiments. Data in the presence of the heavy metals were significantly different from the control (without heavy metals addition), as estimated by Student’s *t*-test (** *p* < 0.01).

It has to be highlighted that, under physiological conditions, OCTN1 is involved in the non-quantal release of acetylcholine from cells in the so-called non-neuronal cholinergic system [19,51,52,53]. Therefore, a different transport assay was used in which the efflux of radiolabelled acetylcholine was measured from proteoliposomes, mimicking the cell release [21] (Figure 8A).

Interestingly, the OCTN1 efflux of radiolabelled acetylcholine was inhibited by the addition of heavy metals in the external environment (trans-inhibition) (Figure 8B). These results indicate that the presence of extracellular metals may also impair, besides the uptake of physiological compounds or cationic drugs, the non-quantal release of acetylcholine, thus impairing its anti-inflammatory action.

Indeed, besides its well-known role as a neurotransmitter, acetylcholine plays pleiotropic functions in non-neuronal tissues, including airways, the intestine, skin, heart, skeletal muscle, placenta, urogenital tract, pancreas islets, and others [29,53,54]. In these districts, acetylcholine is involved in modulating cell proliferation and differentiation, cell–cell communication, and inflammation. In good agreement, the link between OCTN1 and inflammatory diseases has been demonstrated for a long time [55,56]. In good agreement, the gene encoding for OCTN1 maps in the IBD 5 (Inflammatory Bowel Disease) locus on chromosome 5, which has been linked to susceptibility to Crohn’s disease, ulcerative colitis, and rheumatoid arthritis; moreover, a natural mutation of OCTN1, namely L503F, is found in Crohn’s disease patients. The mutant protein shows an impaired ability to release acetylcholine [57], the impaired uptake of carnitine [24] and ergothioneine, and a fungi metabolite with an antioxidant role [58,59]. It is noteworthy that the presence of chronic inflammation is nowadays considered one of the main drivers for cell senescence and ageing, further highlighting the importance of the molecules and macromolecules responsible for handling oxidative stress in cells, which could also be induced by heavy metals. 

### 2.6. OCTN1 as a Meeting Point Between Humans and Fish

**Figure 9:**
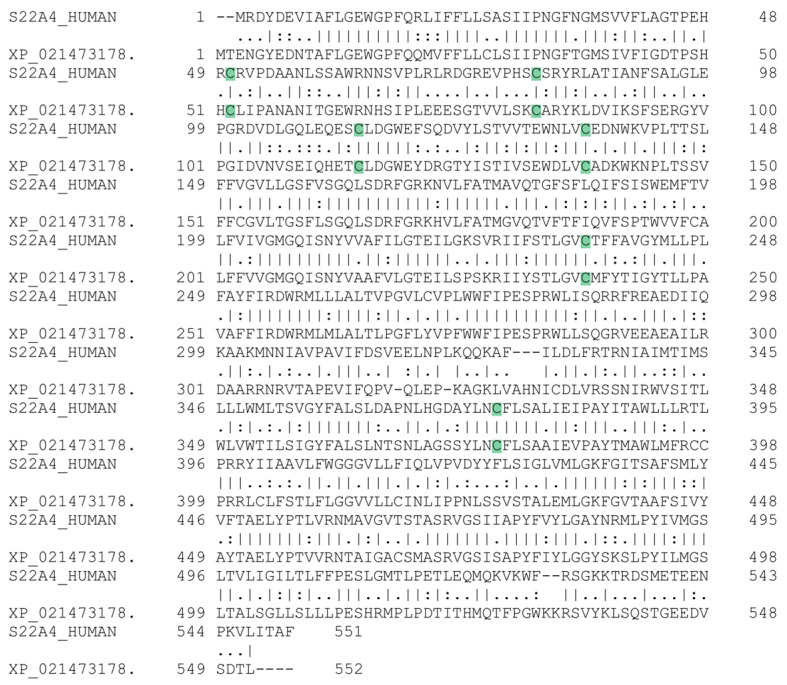
Sequence alignment between human OCTN1 and a possible ergothioneine transporter found in *Oncorhynchus mykiss*. The conserved cysteine residues are highlighted in green. The sequences were taken from UniProt, and the alignment was performed with EMBOSS Needle.

Finally, in a broader environmental context, it has to be highlighted that the orthologue *Oncorhynchus mykiss* has 6 Cys residues conserved with OCTN1 (Figure 9). 

This suggests a common inhibitory mechanism among fish and human isoforms of OCTN1, further demonstrating the wide effects of heavy metals in different species by interacting with a similar target. 

## 3. Materials and Methods

### 3.1. Materials

1,4 Dithioerythritol (DTE), n-dodecyl D-β maltoside (DDM), N-Lauroylsarkosine sodium salt (Sarkosyl), Amberlite XAD-4, Octaethylene glycol monododecyl ether (C_12_E_8_), BIS-Tris, Sephadex G-75, L-α-Phosphatidylcholine, Cholesterol, Imidazole, Tetramethylammonium chloride (TEA), Acetylcholine chloride (Ach), Chromium (II) chloride (CrCl_2_), Zinc chloride (ZnCl_2_), L-Cystein, Mercury (II) chloride (HgCl_2_), Lead nitrate (Pb(NO_3_)_2_), Cadmium chloride (CdCl_2_), Hemicholinium-3, Adenosine-5′-Triphosphate disodium salt, and His-select resin were purchased from Merck Life Science (Milan, Italy); Acetylcholine iodide [acetyl-^3^H]-Acetylcholine was obtained from Perkin-Elmer; [ethyl-1-^14^C]-TEA was obtained from American Radiolabeled Chemicals; and PD-10 Desalting Column was obtained from GE Healthcare UK LTD. All the other reagents were of analytical grade. 

### 3.2. Over-Expression of OCTN1 WT and C-Less Mutant

The over-expression of OCTN1 wild type (WT) and C-less mutant proteins, as previously described in [17], has been performed in *E. coli* Rosetta(DE3)pLysS, as described in [60,61]. 

### 3.3. Purification of WT and Mutant OCTN1 Transporter

**Figure 10:**
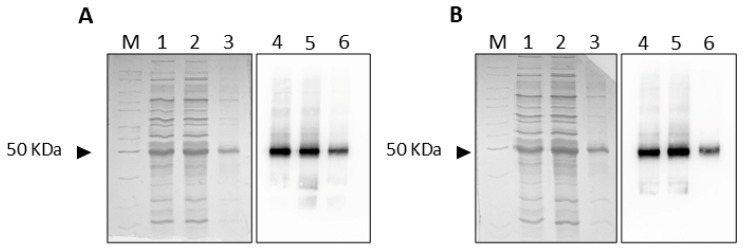
SDS-PAGE and Western blot analysis. In (**A**), the purified WT-OCTN1 is shown, and in (**B**), the purified C-less OCTN1 is shown. Lanes 1 and 4: cell lysates of the over-expressed proteins. Lanes 2 and 5: supernatant of solubilized proteins before purification. Lanes 3 and 6: purified proteins, as described in Section 3. Lane M: molecular mass marker (Thermo Fisher Scientific, Milan Italy—product code: 26614). Samples were separated by SDS–PAGE and stained using Coomassie Blue (**Left**) or detected by using Anti-His antibody in the Western blotting procedure (**Right**).

The WT (Figure 10A) and C-less (Figure 10B) OCTN1 proteins were purified as previously described [23] with some modifications. In brief, the insoluble fraction of 4 mL (OCTN1-WT) or 20 mL (OCTN1-C-less) of cell lysate was washed with 2 mL of Tris/HCl 0.1 M (pH 8.0) and centrifuged at 12,000× g for 5 min at 4 °C. The pellet was solubilized with 100 µL of 0.1 M DTE, 54 µL of 10% sarkosyl, and 400 µL of 8 M Urea. Then, 546 µL of a buffer containing 10% glycerol, 200 mM NaCl, 20 mM Tris/HCl (pH 8.0), and 0.1% sarkosyl were added. The resulting mixture was centrifuged at 12,000× *g* for 10 min at 4 °C. The supernatant was applied onto a column filled with His-select Ni^2+^-chelating affinity gel (0.7 cm diameter, 5.2 cm height) pre-conditioned with 8 mL of a buffer containing 10% glycerol, 0.1% sarkosyl, 200 mM NaCl, and 20 mM Tris/HCl (pH 8.0). After 1 h of incubation at 4 °C, the elution was performed with 5 mL of a buffer containing 10% glycerol, 200 mM NaCl, 20 mM Tris/HCl (pH 8.0), 0.05% DDM, and 1 mM DTE, and then 3 mL of the same buffer containing 10 mM imidazole. OCTN1 was eluted with 4 mL of 200 mM imidazole added with 0.05% DDM, 1 mM DTE, 10% glycerol, and 20 mM Tris/HCl (pH 8.0). The eluate (2 mL) containing OCTN1 was applied onto a Desalting Column (PD-10), previously equilibrated with 25 mL of a solution composed of 0.05% DDM, 1 mM DTE, and 10% glycerol, 20 mM Tris/HCl (pH 8.0). The elution of OCTN1 was obtained by applying 3.5 mL of the same solution described above. The first 1.5 mL was discarded, and the following 2 mL, containing OCTN1, was collected and used for reconstitution in proteoliposomes. 

### 3.4. Liposome Preparation

To remove calcium phosphate from phospholipids, 3 mM EDTA was added to 10% egg yolk phospholipids and incubated for 15 min at room temperature. Then, chloroform was added in a 1:1 ratio with phospholipids, and the solution was centrifuged for 15 min at 12,000× *g*, at 4° C, using a fixed angle rotor. The supernatant containing clean phospholipids in chloroform was evaporated by a rotavapor at 40° C. Then, 25 mg of cholesterol was added to 100 mg phospholipids and dissolved with chloroform. After incubation, under rotatory stirring (30 °C 15 min 750 rpm), the solution was dried using a rotavapor. The lipid film was resuspended in water (10% final concentration), and single-bilayer liposomes were prepared by two sonication cycles of 1 min (1 pulse ON and 1 pulse OFF, 40 W) with a Vibracell VCX-130 sonifier (VWR, Milan, Italy) [62].

### 3.5. Reconstitution of OCTN1 Transporter into Liposomes

After desalting, the protein was reconstituted in liposomes. The composition of the initial mixture used for reconstitution was as follows: 50 µg of protein (or desalt buffer in liposomes without the incorporated protein), 80 µL of 10% C_12_E_8_, 120 µL of 10% egg yolk phospholipids with 25% cholesterol in the form of sonicated liposomes, 16 mM ATP disodium salt, and 5 mM BisTris/HCl (pH 7.5) in a final volume of 700 µL. The purified OCTN1 (WT or C-less mutant) was inserted in the liposomal membrane using the batch-wise technique. This procedure used 0.5 g of Amberlite XAD-4 to remove the detergent from mixed micelles consisting of proteins, phospholipids and detergent at 23 °C for 40 min at 1200 rpm, as previously described [63].

### 3.6. Transport Measurements

Proteoliposomes and liposomes (600 μL) were applied onto a Sephadex G-75 (Merck Life Science, Milan, Italy) column (0.7 cm diameter × 15 cm height) pre-equilibrated with 5 mM BIS-Tris/HCl (pH 8.0) and eluted with 600 μL of the same buffer. Samples of 100 μL each were prepared from the 600 μL pool. The transport was initiated by [^14^C]-TEA or [^3^H]-Ach at the concentration of 0.1 mM to the proteoliposome samples and stopped after 60 min according to the stop-inhibitor method [57]. In the blank samples, the inhibitor (1 mM Hemicholinium-3) was added at time zero. After the transport time, each sample was applied onto a Sephadex G-75 column (0.6 cm diameter × 8 cm height) and pre-equilibrated with 50 mM NaCl to remove the radioactivity not taken up. Proteoliposomes were eluted with 50 mM NaCl (1 mL), added to 3 mL Pico-Fluor Plus, vortexed, and counted. The radioactivity in the blank samples was used to subtract background radioactivity from the protein-associated one. 

For efflux measurements, proteoliposomes (600 µL) were preloaded with radioactive 0.1 mM [^3^H]-Ach for 120 min. External compounds were removed by another passage of the proteoliposomes through Sephadex G-75. The efflux measurement was started by adding 100 µM of the indicated heavy metals. The transport was stopped after 60 min by using the stop-inhibitor method, as previously reported. The percentage of residual activity with respect to the control was reported. 

### 3.7. Other Methods

The amount of purified protein was estimated from stain-free 12% SDS–PAGE gels using the Chemidoc imaging system equipped with Image lab software (Bio-Rad Laboratories srl, Milan, Italy) in absolute quantification using BSA as a standard. Recombinant OCTN1 WT and C-less proteins were immuno-detected using the Monoclonal Anti-polyhistidine–Peroxidase antibody 1:10,000 after 1 h incubation at room temperature. The reaction was detected by Electro Chemi Luminescence (ECL). The human OCTN1 homology model was obtained using the 3D structure of OCT3 (PDB ID: 7ZH0) by Modeller 10.2 [23,64]. This structure was analyzed by the MIB2 server to predict a potential Cd^2+^ binding site [65].

Sequence alignment was performed with EMBOSS Needle and Protein sequences, which were taken from the GenBank database [66,67]. 

Illustration sketches were prepared using https://www.biorender.com/ (accessed on 22 October 2024).

### 3.8. Data Analysis

All experimental data were derived from the mean of three independent experiments, and results were expressed as means ± SD. IC_50_ values were derived from data fitting the IC_50_ equation using Grafit v 5.0.13 software (Erithacus Software (v 5.0.13), East Grinstead, UK).

Comparisons between the two groups were performed with the two-tailed Student’s unpaired *t*-test for * *p* < 0.05 and ** *p* < 0.01. 

## 4. Conclusions 

In this work, the human transporter OCTN1 was exploited as a benchmark for deciphering the molecular mechanism of interactions with heavy metals potentially released by aquatic microplastics. It has to be stressed that the collected results may have great relevance in the context of environmental health because OCTN1 emerged with vertebrates and, hence, orthologues are present in humans and fishes. Due to the biomagnification phenomena, the effects of heavy metals could not appear in a short time as acute effects and could be only appreciable after a long period of chronic exposure. Therefore, it is of paramount importance to understand the action and mechanisms of heavy metals to prevent or treat the consequences of long-term exposure. It is noteworthy that the mechanisms described for OCTN1 may be common to other biological targets in humans and in other living organisms, further enlarging the collection of negative effects ascribed to heavy metals exposure. As an example, within the SLC22 family, OCT members have conserved cysteine residues in the same position of the metal-binding site of OCTN1 [68]. In this scenario, it can be hypothesized as a beneficial effect to use some antioxidants, such as cysteine or acetyl-cysteine, in individuals subjected to heavy metal exposure. Intriguingly, the negative effects of heavy metals can be worsened by interactions with microplastics and nanoplastics, whose risks for biota are already well known [4]. Taken together, the described data highlight two key and complementary needs in this research field: (i) experimental tools devoted to studying the effects of pollutants related to microplastic spread and (ii) improvements in the identification of novel targets across different species to reduce the risks and face future challenges.

## Figures and Tables

**Table 1:** The classification of microplastics according to their size.

Particles Size (mm)	Classification
>5 mm	Macroplastics
Between 5 mm and 0.3 mm	Microplastics
<0.3 mm	Nanoplastics

**Table 2:** Toxicity level of metals tested according to ATSDR. High toxicity (+++), Low toxicity (+).

Heavy Metals	Level of Toxicity	Identifier
Mercury	+++	https://www.atsdr.cdc.gov/toxprofiles/tp46.pdf (accessed on 21 October 2024)
Cadmium	+++	https://www.atsdr.cdc.gov/toxprofiles/tp5.pdf (accessed on 21 October 2024).
Zinc	+	https://www.atsdr.cdc.gov/toxprofiles/tp60.pdf (accessed on 21 October 2024).
Lead	+++	https://www.atsdr.cdc.gov/toxprofiles/tp13.pdf (accessed on 21 October 2024).
Chromium	+++	https://www.atsdr.cdc.gov/toxprofiles/tp7.pdf (accessed on 21 October 2024).

**Table 3:** IC_50_ values (µM) calculated from dose–response analysis of OCTN1 WT in the presence of HgCl_2_, CdCl_2_, ZnCl_2_, Pb(NO_3_)_2_, and CrCl_2_ on [^14^C]-TEA uptake.

Heavy Metals	IC_50_ (µM)
HgCl_2_	121 ± 7
CdCl_2_	56 ± 4
ZnCl_2_	57 ± 17
Pb(NO_3_)_2_	90 ± 19
CrCl_2_	170 ± 15

## Data Availability

The data presented in this study are available on request from the corresponding author.
